# Some Early Reports

**Published:** 1921-02-26

**Authors:** R. Morrison Smith

**Affiliations:** Secretary and Cashier. Glasgow Royal Infirmary


*To the Editor of* The Hospital.

### "Some Early Reports."

*To the Editor of* The Hospital.

Sir,—On page 467 of your issue dated February 19, 1921, I observe that you state, "The working class contributed £14,368 [to the Glasgow Royal Infirmary] during the year." This should read £14,368 *more*. You will see from the enclosed report, the employees' subscriptions amounted to £30,933 in 1920, as compared with £16,565 in 1919, being an increase over 1919 of £14,368, out of a total increased revenue of £28,923.

Kindly note this correction in your next issue.

Yours faithfully,

R. Morrison Smith, Secretary and Cashier.

Glasgow Royal Infirmary.

[We have much pleasure in correcting our error and doing justice to the noteworthy increase here recorded.—Ed. The Hospital.]

